# Kremen1-induced cell death is regulated by homo- and heterodimerization

**DOI:** 10.1038/s41420-019-0175-5

**Published:** 2019-05-01

**Authors:** Iffat Sumia, Alessandra Pierani, Frédéric Causeret

**Affiliations:** 1 Institute of Psychiatry and Neuroscience of Paris, INSERM UMR-S 1266, Université de Paris, Paris, France; 2 Imagine Institute, Université de Paris, Paris, France

**Keywords:** Apoptosis, Cancer, Cell signalling, Cell death

## Abstract

In multicellular organisms, cell death pathways allow the removal of abnormal or unwanted cells. Their dysregulation can lead either to excessive elimination or to inappropriate cell survival. Evolutionary constraints ensure that such pathways are strictly regulated in order to restrain their activation to the appropriate context. We have previously shown that the transmembrane receptor Kremen1 behaves as a dependence receptor, triggering cell death unless bound to its ligand Dickkopf1. In this study, we reveal that Kremen1 apoptotic signaling requires homodimerization of the receptor. Dickkopf1 binding inhibits Kremen1 multimerization and alleviates cell death, whereas forced dimerization increases apoptotic signaling. Furthermore, we show that Kremen2, a paralog of Kremen1, which bears no intrinsic apoptotic activity, binds and competes with Kremen1. Consequently, Kremen2 is a very potent inhibitor of Kremen1-induced cell death. Kremen1 was proposed to act as a tumor suppressor, preventing cancer cell survival in a ligand-poor environment. We found that *KREMEN2* expression is increased in a large majority of cancers, suggesting it may confer increased survival capacity. Consistently, low *KREMEN2* expression is a good prognostic for patient survival in a variety of cancers.

## Introduction

In multicellular organisms, a tight control of cell survival and death is crucial to ensure normal development, tissue homeostasis, and viability. This is achieved through the combinatorial action of multiple intracellular pathways regulated by various extracellular cues and cognate transmembrane receptors. Many of the molecular players involved in survival/death signal transduction are not restricted to such a function but often, also participate in distinct, unrelated, physiological processes. This is perhaps best exemplified by the case of dependence receptors.

Dependence receptors are a group of receptors that differ in the cellular response they initiate upon ligand binding but share the ability to trigger cell death upon ligand deprivation^1^. Cells expressing dependence receptors therefore depend on ligand availability for their survival. To date, >15 dependence receptors have been characterized, among which DCC, Unc5h, Cdon, c-Kit, PlexinD1, p75^NTR^, and Notch3^2–8^ figure. The importance of “positive” canonical signaling through these receptors during mammalian embryonic development is well established. Over the past decade, multiple studies focusing on the in vivo relevance of dependence receptors “negative” pro-apoptotic signaling led to the proposal that they behave as tumor suppressors^9^. Thus, the death-inducing activity of dependence receptors would favor the elimination of cells (metastatic for instance) migrating away from their normal ligand-rich environment. Conversely, a loss of dependence receptor function resulting from decreased expression, somatic mutation or gain of ligand expression, may confer a selective advantage to cancer cells and allow tumor growth in a normally non-permissive environment^1^. Consistently, many dependence receptors were shown to be downregulated and their ligands upregulated in cancers thus favoring cancer cells survival^9^.

More recently, the possibility to target dependence receptor signaling in order to selectively favor cancer cell death has emerged as a valid therapeutic strategy. Netrin-1 receptors DCC and Unc5h are the most studied dependence receptors. Netrin-1 interference using a soluble recombinant domain of DCC was shown to induce cancer cell death in vitro and in vivo^10–12^. In addition, a Netrin-1 monoclonal antibody was proven an efficient anticancer agent in mice^13^.

In a previous study^14^, we identified the transmembrane receptor Kremen1 (Krm1) as a novel dependence receptor. In the absence of its ligand Dickkopf1 (Dkk1), a well-characterized Wnt-antagonist^15^, Krm1 is able to induce Caspase-3 activation, a hallmark of apoptosis^16^, independent of Wnt canonical signaling. Such a behavior is neither shared with Krm1’s non-mammalian homologs, nor with its paralog Krm2. Consistent with an implication of Krm1 apoptotic signaling dysregulation in cancer, human gene *KREMEN1* expression tends to be decreased in a variety of tumors and cancer cell lines^14,17^. In addition, we demonstrated that somatic mutations found in cancer patients can affect Krm1 apoptotic activity^14^.

In this study, we tackle the issue of the regulation of Krm1 apoptotic activity. We demonstrate that Krm1 homodimerization is required for cell death induction, whereas heterodimer formation with its paralog Krm2 prevents pro-apoptotic signaling. Consistently, we found that *KREMEN2* expression is increased in a large majority of cancers and that high *KREMEN2* expression in tumors is linked to a poor outcome in multiple cancers.

## Results

### Krm1 dimerizes through its extracellular domain

**Fig. 1:**
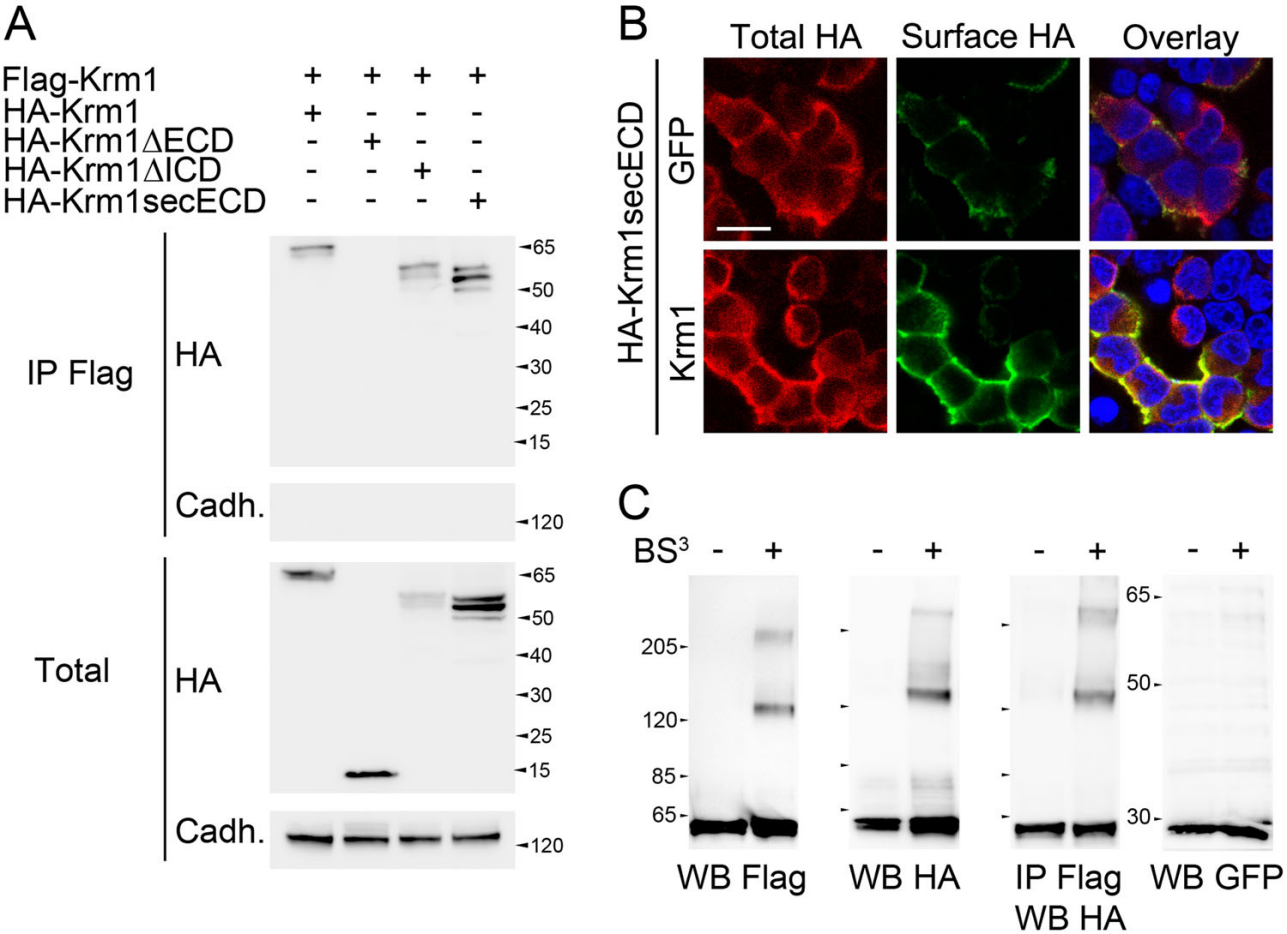
Krm1 homodimerizes through its extracellular domain. **a** Lysates from HEK293T cells transfected with Flag-Krm1 and HA-tagged full-length or truncation mutants of Krm1 lacking either the extracellular domain (ΔECD), the intracellular domain (ΔICD), or the transmembrane and intracellular domains (secECD) and subjected to anti-Flag immunoprecipitation followed by western blot using an anti-HA. An anti-panCadherin was used as control. Straight western blots on lysates are shown on the bottom panels. **b** Surface (green) and total (red) HA immunostaining of HEK293T cells transfected with HA-Krm1secECD together with either Green Fluorescent Protein (GFP, top lane) or full-length Krm1 (bottom lane). Scale bar: 10 µm. **c** Western blots and immunoprecipitation following extracellular proteins crosslinking with BS^3^ of HEK293T cells transfected with HA-Krm1 and Flag-Krm1

The well-characterized dependence receptors p75^NTR^, DCC, and Unc5h are known to multimerize in a ligand-dependent manner, which inhibits their pro-apoptotic activity^18,19^. In order to determine whether the newly identified dependence receptor Krm1 is also subjected to multimerization, we first transfected HEK293T cells with plasmids encoding Hemagglutinin (HA)- and Flag-tagged versions of Krm1. Cells extracts were collected after 24 h and subjected to immunoprecipitation using an anti-Flag antibody. Western blot indicated that HA-Krm1 is detected in the immunoprecipitates, whereas other transmembrane proteins such as Cadherins are not (Fig. 1a). Using truncated versions of HA-Krm1, lacking the intracellular domain (ΔICD) or the extracellular domain (ΔECD) we found that co-immunoprecipitation requires the extracellular domain of Krm1. Furthermore, membrane anchoring appeared dispensable since a secreted ectodomain (secECD) retains its ability to interact with full-length Krm1 (Fig. 1a).

In order to visualize Krm1 extracellular domain multimerization at the cell surface, we then transfected HA-tagged Krm1secECD together with plasmids coding for either GFP or untagged full-length Krm1. The cells were then subjected to surface and total HA immunostaining, allowing us to distinguish between the amounts of secECD produced by the cells and sequestered at the cell surface. We observed a dramatic increase in surface staining in the presence of Krm1 compared with GFP (Fig. 1b), indicating that HA-Krm1-secECD is retained at the cell surface by full-length Krm1.

We further tested whether the interaction between Krm1 molecules consists in dimerization and/or oligomerization. To this end, we treated HEK293T cells transfected with both Flag- and HA-tagged Krm1 and treated them with the non cell-permeant crosslinking reagent bis(sulfosuccinimidyl)suberate (BS^3^) just prior to lysis. Straight western blots of the lysates using either HA or Flag antibodies showed that in addition to 60 kDa Krm1 monomers, crosslinking unraveled a sharp band around 120 kDa and a weaker and fuzzier one above 200 kDa (Fig. 1c). By contrast, GFP blots were identical in the presence of absence of BS^3^. Immunoprecipitation with an anti-Flag followed by western blot with an anti-HA gave the same pattern as straight blots (Fig. 1c), demonstrating that the 120 kDa band contains HA-Krm1/Flag-Krm1 dimers. The 200 kDa band also contains HA-Krm1 and Flag-Krm1 and could be interpreted either as a receptor complex containing two molecules of Krm1 together with one or several partners (whose combined molecular weight would be in the range of 100 kDa), or as a trimer subjected to important posttranslational modification (e.g., glycosylation).

Taken together, these experiments led us to conclude that Krm1 homodimerizes through its extracellular domain.

### Krm1 dimerization regulates apoptotic signaling

**Fig. 2:**
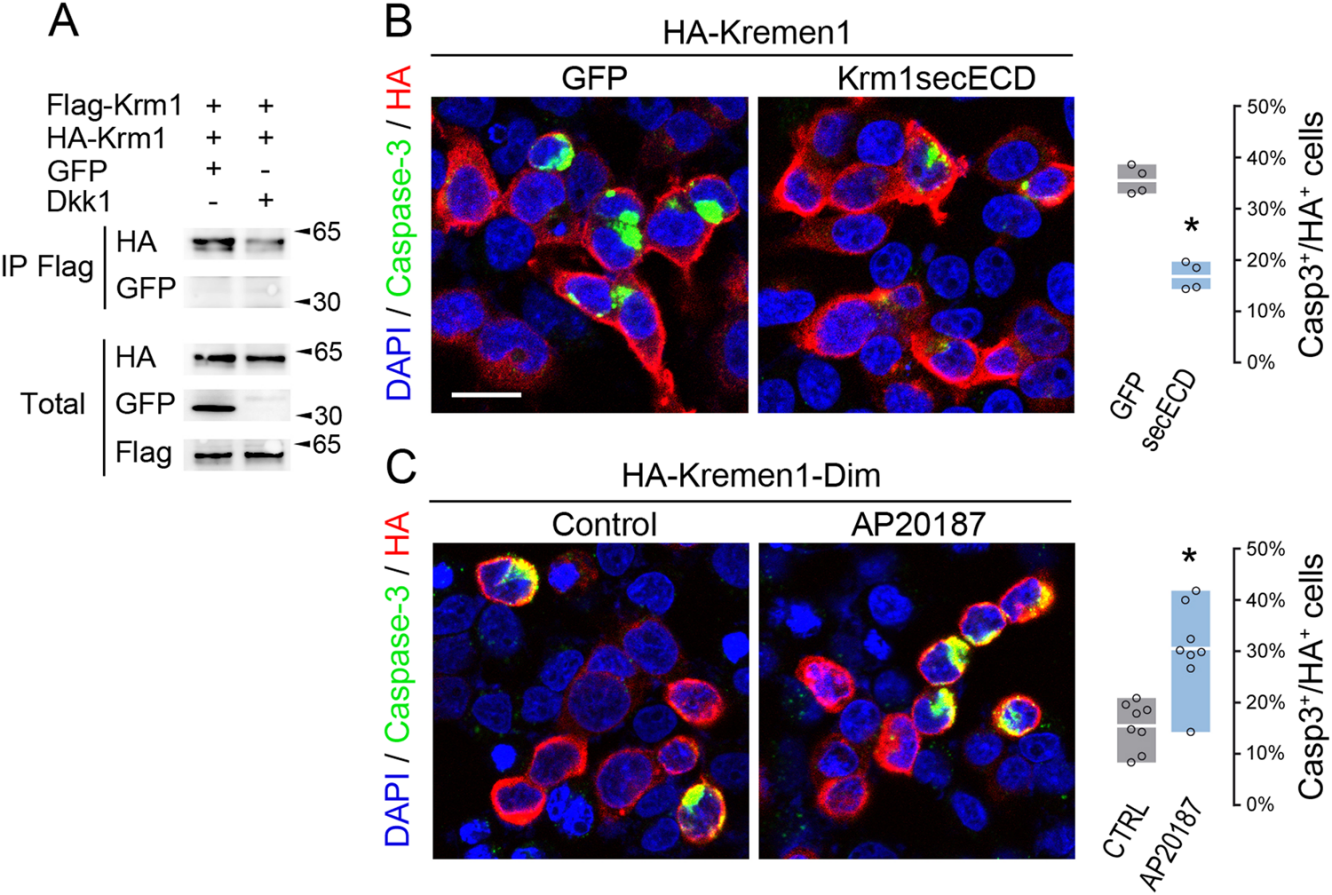
Krm1 dimerization regulates apoptotic signaling. **a** Lysates from HEK293T cells transfected with Flag-Krm1 and HA-Krm1 together with either GFP or Dkk1 and subjected to anti-Flag immunoprecipitation followed by western blot using an anti-HA. An anti-GFP was used as control. Straight western blots on lysates are shown on the bottom panels. **b** Cleaved Caspase-3 (green) and HA (red) immunostaining of HEK293T cells transfected with HA-Krm1 and either GFP or Krm1secECD. Nuclei are stained with DAPI (blue). The histogram indicates the proportion of Caspase-3^+^ cells among HA^+^ cells (average ± sd). *p < 0.001 using Student’s *t-*test. Scale bar: 10 µm. **c** Cleaved Caspase-3 (green) and HA (red) immunostaining of HEK293T cells transfected with a Krm1 construct containing FK506 binding domains (HA-Kremen1-Dim) treated or not with 50 nM of the dimerizing drug AP20187. The histogram indicates the proportion of Caspase-3^+^ cells among HA^+^ cells (average ± sd). *p < 0.001 using Student’s *t*-test

The homodimerization of p75^NTR^, DCC, and Unc5h dependence receptors was previously shown to be increased upon ligand binding, thus preventing the apoptotic activity of the receptors^18,19^. In order to test whether this is also the case for Krm1, we transfected HEK293T cells with HA-Krm1, Flag-Krm1 and either GFP or Dkk1. As illustrated Fig. 2a, immunoprecipitation experiments indicated that contrary to other dependence receptors, Krm1 dimerization decreases in the presence of its ligand Dkk1.

As Dkk1 binding to Krm1 inhibits both apoptotic signaling^14^ and dimerization, we tested whether these two processes are linked, as is the case for p75^NTR^, DCC, and Unc5h^18,19^ by interfering with Krm1 dimerization. To this end, we co-transfected HEK293T with plasmids encoding Krm1 and either GFP or the secECD of Krm1, which is able to interact with full-length Krm1 but lacks apoptotic activity^14^. We assessed the consequences on apoptosis induction by performing immunostaining to visualize Caspase-3 activation. Measurement of the number of apoptotic cells indicated a twofold decrease in the presence of the secECD compared with GFP (Fig. 2b). We then implemented the reverse experiment, consisting in forcing Krm1 dimerization. This was achieved through the insertion of two FK506 binding domains in the coding sequence of Krm1. Multimerization could then be induced by the addition of the chemical compound AP20187, a bivalent FK506 derivative. Such a system was previously used successfully to induce the multimerization of p75^NTR^, DCC, and Unc5h dependence receptors^18,19^. As illustrated Fig. 2c, forced Krm1 dimerization resulted in a significant increase in apoptotic signaling. These results support a model in which Krm1 dimerization induces cell death.

### Kremen2 negatively regulates Krm1 through heterodimerization

Krm1 and its paralog Krm2 share the ability to bind Dkk1 and inhibit Wnt signaling, both processes previously shown to rely on the extracellular domain^20^. As Krm1 dimerization also requires the ECD, we wondered whether Kremen2 could interfere with Krm1 through heterodimerization.

**Fig. 3:**
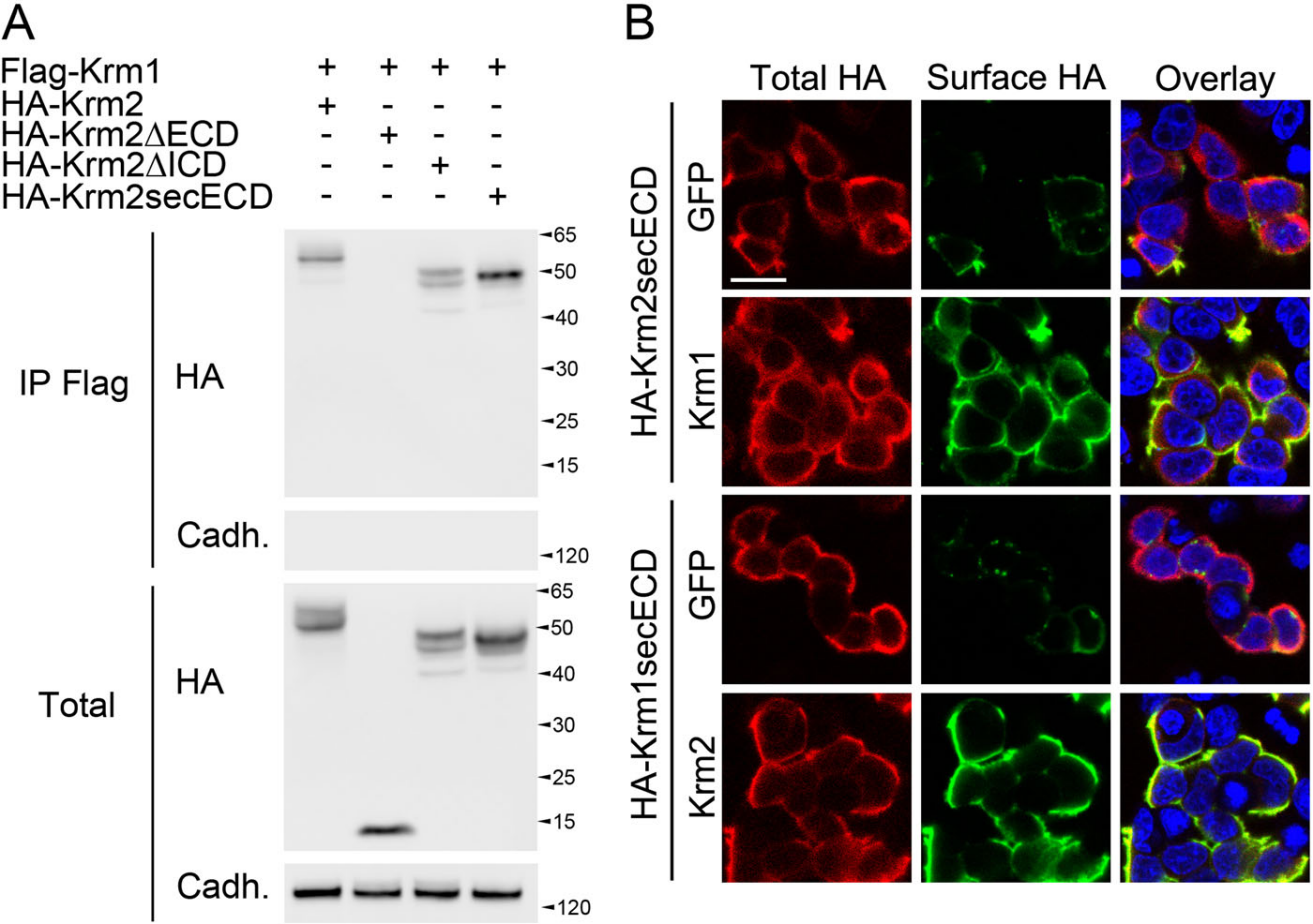
Krm2 interacts with Krm1. **a** Lysates from HEK293T cells transfected with Flag-Krm1 and HA-tagged full-length or truncation mutants of Krm2 lacking either the extracellular domain (ΔECD), the intracellular domain (ΔICD), or the transmembrane and intracellular domains (secECD) and subjected to anti-Flag immunoprecipitation followed by western blot using an anti-HA. An anti-panCadherin was used as control. Straight western blots on lysates are shown on the bottom panels. **b** Surface (green) and total (red) HA immunostaining of HEK293T cells transfected with HA-Krm2secECD together with either GFP (first lane) or full-length untagged Krm1 (second lane), or with HA-Krm1secECD together with either GFP (third lane) or full-length untagged Krm2 (fourth lane). Scale bar: 10 µm

We first performed co-immunoprecipitation experiments on protein extracts of cells transfected with HA-Krm1 and Flag-Krm2. We found that Krm2 can be detected from Krm1 immunoprecipitates, suggesting that the two proteins interact (Fig. 3a). Consistent with our previous findings, we found that the extracellular domain of Krm2 is necessary and sufficient for the interaction with Krm1. We then tested the ability of Krm1 and Krm2 to bind each other in living cells by performing surface staining experiments. As indicated Fig. 3b, we found that both Krm1 and Krm2 were able to retain the secreted ECD of their paralog at the cell surface. These data led us to conclude that Krm1 and Krm2 heterodimerize.

**Fig. 4:**
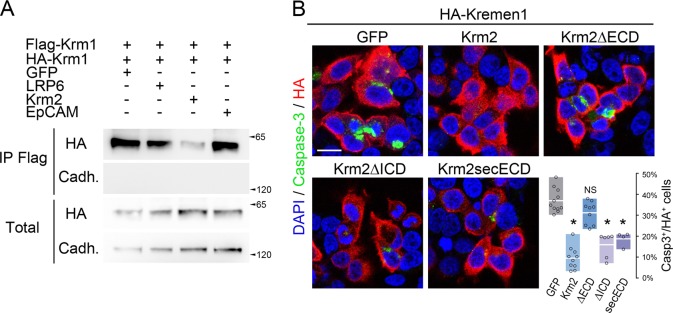
Krm2 inhibits Krm1 dimerization and apoptotic signaling. **a** Lysates from HEK293T cells transfected with Flag-Krm1 and HA-Krm1 together with either GFP, LRP6, Krm2, or EpCAM and subjected to anti-Flag immunoprecipitation followed by anti-HA western blot. An anti-panCadherin was used as control. Straight western blots on lysates are shown on the bottom panels. **b** Cleaved Caspase-3 (green) and HA (red) immunostaining of HEK293T cells transfected with HA-Krm1 and either GFP or Krm2 constructs. The histogram represents the proportion of Caspase-3^+^ cells among HA^+^ cells (average ± sd) following immunostaining. *p < 0.001 using Student’s *t-*test. Scale bar: 10 µm

We next assessed to which extent could the presence of Krm2 inhibit or potentiate Krm1 dimerization. We first performed a competition experiment consisting in the transfection of HEK293T cells with HA-Krm1, Flag-Krm1 and either GFP or Krm2 followed by a co-immunoprecipitation between Flag-Krm1 and HA-Krm1. We found that the addition of Krm2 strongly reduced Krm1 dimerization (Fig. 4a). By contrast, neither LRP6 nor EpCAM, two known partners of Krm1^21,22^ had the same effect. We therefore concluded that Krm2 interaction with Krm1 prevents the ability of the latter to dimerize.

Given our previous findings that the homodimerization of Krm1 is required for its apoptotic signaling and that Krm2 is able to interfere with Krm1 homodimerization, we predicted that Krm2 would antagonize Krm1-induced cell death. We tested this hypothesis by performing Caspase-3 immunostaining on cells transfected with Krm1 and either GFP or Krm2. As illustrated Fig. 4b, we found that Krm2 efficiently silences Caspase-3 activation induced by Krm1. Furthermore, truncation constructs revealed that the ECD of Krm2 is required for such an effect whereas both the ICD and transmembrane domain are dispensable (Fig. 4b).

Taken together, our data demonstrate that Krm1-Krm1 dimers are capable of apoptotic signaling, a process that is negatively regulated by disruption of the complexes upon ligand binding or heterodimerization with Krm2.

### *KREMEN2* is upregulated in cancers

**Fig. 5:**
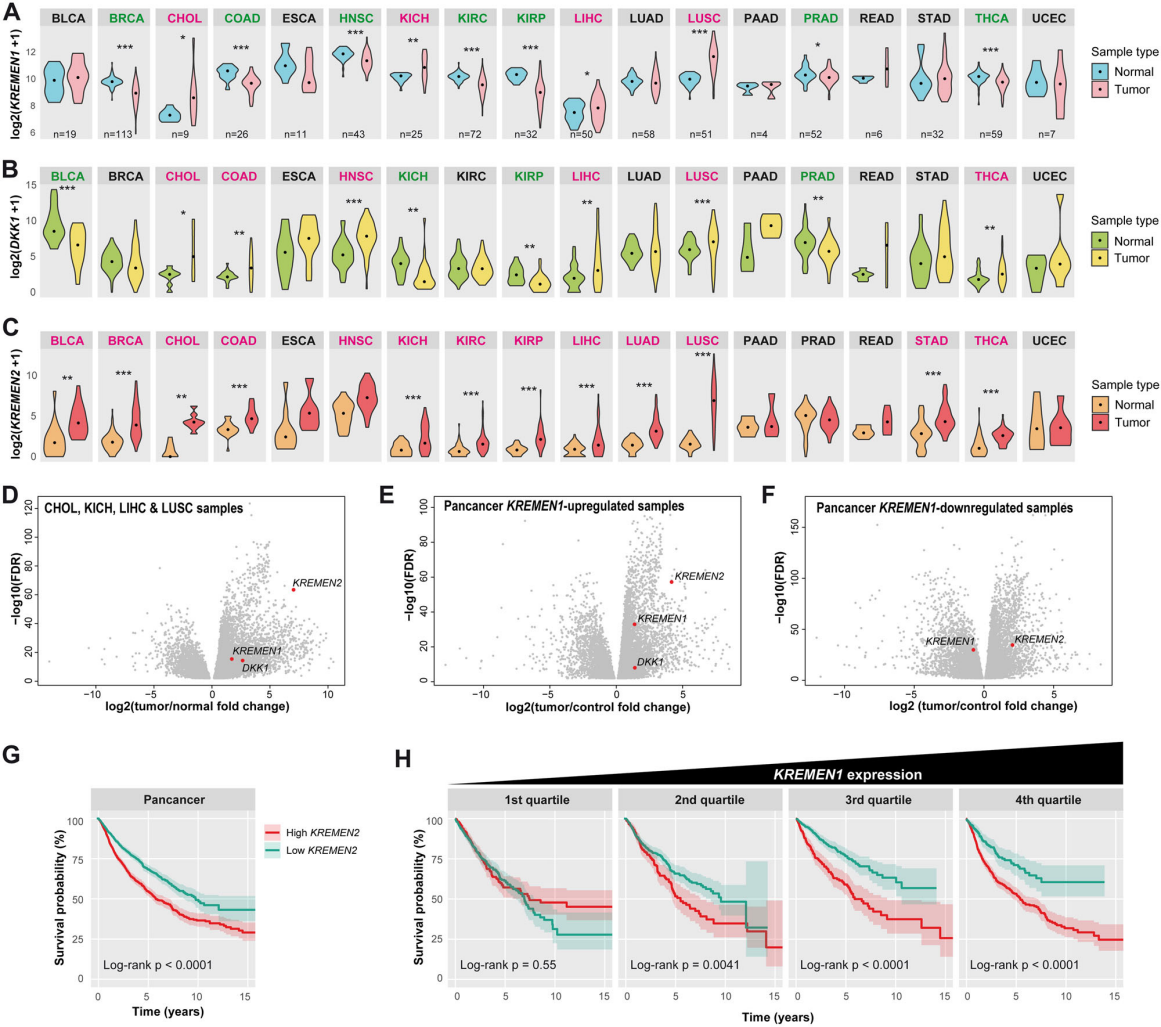
*KREMEN2* is upregulated in cancers. **a**-**c** Violin plots representing the log2 of expression for *KREMEN1* (**a**), *DKK1* (**b**), and *KREMEN2* (**c**) in TCGA tumor and matching normal tissue samples. The abbreviations for cancer types can be found on the *Genomic Data Commons* portal (https://gdc.cancer.gov/) and in Supplementary Table 3. Median expression is indicated by a dot. The number of patients is reported only on the first lane. A color code indicates whether the expression significantly increases (red) or decreases (green) in tumor samples relative to normal tissue from the same patient. *p < 0.05, **p < 0.01, ***p < 0.001 using Wilcoxon test. **d**, **e** Volcano plots representing differentially expressed genes between tumor and normal tissue. *KREMEN1*, *KREMEN2*, and *DKK1* are indicated by a red dot whenever significantly (FDR < 0.01) up- or downregulated. **d** *KREMEN2* is among the most significantly upregulated genes in cancer types in which *KREMEN1* expression is increased. **e** *KREMEN2* is also very significantly upregulated in tumors showing increased *KREMEN1* expression, regardless of the cancer type. **f** Conversely, *KREMEN2* does not stand out among the most upregulated genes in tumors showing decreased *KREMEN1* compared with normal tissue. **g**, **h** Kaplan–Meier curves indicating the survival probability (±confidence interval) of the 25% of patients with the highest *KREMEN2* expression in tumors (red) compared with the 25% with the lowest expression (blue). The log-rank *p*-value is indicated below each graph. **g** Survival curves for patient of all cancer types indicate a significantly better outcome for those with low *KREMEN2* expression in tumors. **h** Survival curves for patient of all cancer type ranked by the level of *KREMEN1* expression in tumors show that the link between low *KREMEN2* expression in tumors and better survival is most significant when *KREMEN1* expression is high

The involvement of dependence receptors in cancers is well documented^1^ and our previous findings suggest that Krm1 acts as a tumor suppressor^14^. We therefore hypothesized that Krm2-mediated Krm1 antagonism could favor the abnormal survival of cancer cells. To begin investigating such a question, we extracted *KREMEN1* and *KREMEN2* as well as *DKK1* expression data from The Cancer Genome Atlas (TCGA). Pairwise comparison between tumor and matching normal tissue in individual patients indicated that *KREMEN1* expression is downregulated in a variety of cancer, especially breast, colon, and kidney renal cell carcinomas (Fig. 5a, see Supplementary Table 3 for abbreviations) as previously reported^14^. However, Krm1 was also upregulated in some cancers and most remarkably in lung squamous cells carcinoma (Fig. 5a).

*DKK1* expression appeared quite variable between and within cancer types (Fig. 5b). In addition, the range of up- or downregulation in tumor versus control tissue was very high compared with *KREMEN1* (not illustrated), indicating that Dkk1 is unlikely to damper Krm1 apoptotic activity in all cancers. By contrast, *KREMEN2* expression was found to be increased in tumor compared with normal tissue in >80% of samples considered, regardless of the cancer type (Fig. 5c). In lung squamous cells carcinoma, the median increase was >10-fold.

In order to identify the genes that are most likely to prevent Krm1-induced cell death in tumors, and evaluate the likelihood of Krm2 and Dkk1 playing such a role, we performed differential gene expression analyses. When considering only the cancer types in which *KREMEN1* is upregulated, we found *KREMEN2* to fall among the most significantly upregulated genes (Fig. 5d and Supplementary Table 4). This was also the case when taking into account only patients that display increased *KREMEN1* expression in tumors, regardless of the cancer type (Fig. 5e and Supplementary Table 5). By contrast, in patients with decreased *KREMEN1* expression in tumors, we found that *KREMEN2* does not stand out among the most significantly upregulated genes (Fig. 5f). These observations indicate that the vast majority of tumors display increased *KREMEN2* expression compared with normal tissue and that the increase is even more pronounced when *KREMEN1* is upregulated, consistent with the idea that Krm2 may promote the abnormal survival of cancer cells through Krm1 antagonism.

Finally, we thought to evaluate the consequences of *KREMEN2* expression in tumors on patient outcome. We computed Kaplan–Meier curves to compare the survival of patients bearing tumors with high (top 25%) or low levels (bottom 25%) of *KREMEN2*. When considering all patients regardless of the cancer type, we found that low *KREMEN2* expression is a factor of good prognosis: 68 ± 3% survival at 5 years versus 54 ± 3% (*p* < 0.0001; Fig. 5g). When cancer types where considered individually, we found a significantly better survival for patients with low *KREMEN2* in bladder carcinoma, kidney clear and papillary cells carcinoma, lung adenocarcinoma and sarcoma (Supplementary Fig. 1a). Furthermore, in order to investigate epistasis between KREMEN1 and KREMEN2, we compared the influence of *KREMEN2* expression on the survival of patients from all cancer cohorts ranked by the level of *KREMEN1* expression in tumor. We found that the association between low *KREMEN2* and better survival increases with the levels of *KREMEN1* expression (Fig. 5h). A similar result was obtained when excluding the abovementioned five cancer types showing a link between low *KREMEN2* and better survival (Supplementary Fig. 1b), indicating that biased sampling cannot account for the observed result. These data therefore suggest that low *KREMEN2* expression has a beneficial effect on patient survival that is not strictly restricted to specific types of cancer but rather depends on the level of *KREMEN1* expression in the tumor. They also support the hypothesis that the mechanism of regulation of Krm1-induced apoptosis through homo- and heterodimerization that we report in this study is of clinical relevance. In this scope, therapeutic strategies aiming at interfering with Krm1 dimerization in order to favor cancer cell death could prove relevant.

## Discussion

We have previously shown that mammalian Krm1 is an efficient inducer of cell death in vitro ^14^. Multiple studies reported in vivo expression of Krm1 in a wide range of tissues during embryogenesis and postnatal life in mice^17,23–26^. These seemingly incompatible observations suggest that Krm1 apoptotic activity is tightly regulated. Several silencing mechanisms have been put forward, including ligand binding, alternative splicing, and targeting by miRNA^14,27^. We now unravel an additional negative regulatory mechanism involving heterodimerization of Krm1 by its paralog Krm2.

We show that Krm2 is a very potent inhibitor of Krm1 apoptotic activity. To our knowledge, this is the first example of a dependence receptor being regulated by a partner in cis. Contrary to soluble ligand (Dkk1) binding, Krm2 antagonism is purely cell autonomous as we failed to detect interactions in trans between Krm1 and Krm2 (unpublished observations). Thus, a Krm2-rich environment, unlike a Dkk1-rich environment, is not sufficient to prevent the death of Krm1-expressing cells. The higher efficiency of Krm2 compared with Dkk1 to inhibit Krm1-induced apoptosis was evident in our assays and could result from differences in binding affinity, availability (membrane confinement vs extracellular diffusion), or protein stability. Although our crosslinking experiments suggest that Krm1 dimerization does not strictly require another partner, we cannot rule out the involvement of additional extracellular players since a >200 kDa complex composed of two molecules of Krm1 and unidentified protein(s) was detected. Our co-immunoprecipitation experiments indicate that both Dkk1 and Krm2 efficiently dissociate Krm1 dimers.

The data we have accumulated in this study led us to propose that Krm1 dimerization is required for apoptosis. Yet, we have previously reported that removal of the extracellular domain renders Krm1 extremely apoptotic^14^, despite the requirement of this domain for dimerization (this study). Although we have no clear explanation for Krm1ΔECD superactivity, we can speculate that the deletion of the extracellular domain induces conformational changes in the intracellular domain that make dimerization no longer required for apoptosis to proceed. Recently, the tumor necrosis receptor superfamily member DR5 was shown to dimerize through its transmembrane domain. Ligand binding to DR5 was proposed to trigger the formation of high-order oligomers able to induce cell death^28^. Interestingly, truncation of DR5 extracellular domain resulted in an increase of apoptosis, suggesting that this domain exerts steric hindrance preventing clusterization of the receptor and subsequent signaling.

Prior to this study, other dependence receptors were shown to form homodimers. For instance, RET, TrkA/C, EphA4, MET, ALK, IGF-1R, and c-Kit are all dependence receptors of the receptor tyrosine kinase family, whose enzymatic activity is induced upon ligand-induced dimerization^29^. Paralogs of PlexinD1 and Notch3 were also shown to homodimerize in a ligand-dependent manner^30,31^, suggesting that regulation of dependence receptors signaling through changes in the multimerization state is perhaps more common than anticipated. Yet, this has been formally demonstrated only for DCC, Unc5, and p75^NTR^^18,19^ although in these cases, opposite to Krm1, dimerization was detrimental to apoptosis induction. Regarding p75^NTR^, biochemical and structural data support a model whereby receptor dimers exist in the absence of ligand. Complex conformational changes induced upon ligand binding, rather than a simple monomer/dimer switch, would then control the recruitment of intracellular effectors and subsequent activation of positive or negative downstream pathways depending on the context^32,33^. To date, the precise cell death subroutine triggered downstream of unliganted Krm1 remains elusive beyond the high level of Caspase-3 activation that is reminiscent of apoptosis^14^. One can speculate that assembly of the signaling cascade only occurs once a Krm1 dimer-specific partner is recruited. The present study lays the first brick in the comprehensive characterization of the molecular events linking receptor dimerization to Caspase-3 cleavage, which will be instrumental to the definitive classification of Krm1-induced cell death.

One of the interests of having unraveled a Krm1 regulatory mechanism involving dimerization through the extracellular domain is that it is easier to target than intracellular pathways in view of therapy. Our experiments involving Krm1secECD provide a proof-of-concept that it is indeed possible to manipulate Krm1 dimerization in order to modulate cell death. This is of special interest given the high expression of *krm1* in most tumors. Krm1 and Krm2 behave very differently in cancers, the former being often downregulated and the later upregulated in the vast majority of tumors, indicating that both genes are unlikely to be regulated by the same pathways. Yet, the link between the two genes seems quite intricate given that *KREMEN2* increased expression is far more pronounced when *KREMEN1* is also upregulated. This supports the hypothesis that they function as antagonists in the regulation of cell survival rather than through the function they share in the modulation of Wnt signaling. Consistently, survival analyses also indicated that low KREMEN2 expression is associated with increased survival specifically in the context of high *KREMEN1* expression.

Our study therefore not only provide insights on the mechanisms controlling the apoptotic activity of Krm1, but also suggests these might be dysregulated in cancers and represent, as such, valid therapeutic targets. One possible strategy to further investigate would be the development of agents (e.g., antibodies or recombinant proteins) able to force Krm1 dimerization or to prevent Krm2-mediated antagonism of Krm1 and thus restore normal levels of death in cells with abnormal survival capacities such as cancer cells.

## Materials and methods

### Expression constructs

Plasmids encoding Krm1, Krm2, Dkk1, Lrp6, EpCAM, as well as truncated versions of Krm1 and Krm2, were made in the pCAG-IRES-EGFP vector as described previously^14^. HA or Flag tags were inserted after the signal peptide sequence. In some instances, we used pCS2-Krm1 expression vectors to obtain lower expression levels, to decrease transcriptional squelching, which was sometimes observed when co-transfecting several pCAG vectors, or to avoid the bi-cistronic expression of GFP. For induced dimerization experiments, two FKBP homodimerizer domains were inserted in between the transmembrane and intracellular domains of Krm1 in the pC_4_M-F_v_2E vector (Clontech).

### Cell culture and transfection

HEK293T cells were cultured in Dulbecco’s modified Eagle’s medium supplemented with 10% fetal bovine serum and penicillin/streptomycin. Cells were either seeded on glass coverslips previously coated with poly-l-lysine (for immunostaining experiments) or directly on plastic dishes (for western blot experiments). Transfection was performed for 4 h in Optimem with 2 µL Lipofectamine 2000 (Invitrogen) and 1 µg total DNA per 15 mm well (0.2–0.5 µg DNA of pCAG constructs ; 0.5–1 µg of pCS2 or pC_4_M-F_v_2E constructs; pBluescript KS+ was used to complete whenever necessary). For induced dimerization experiments, AP20187 (Clontech) was used at 50 nM.

### Western blotting

Twenty-four hours after transfection, cells were lysed in a buffer composed of 25 mM Tris pH7.4, 150 mM NaCl, 5 mM EDTA, 1% Triton-X100, 10% Glycerol and cOmplete Protease Inhibitor Cocktail (Sigma). For crosslinking experiments, cells were rinsed in phosphate-buffered saline and incubated on ice for 30 min in the presence or absence of 0.2 mM BS^3^ (Thermo Fisher) prior to lysis. Immunoprecipitations were carried out using 200–500 µg total protein extract, 1–2 µL (corresponding to 1–2 µg) of rabbit anti-Flag (Sigma) or mouse anti-HA (16B12, Convance) and 5–10 µL Dynabeads proteinG (Invitrogen). Western blots were revealed using the following primary antibodies: mouse anti-HA (16B12, Convance, 1:4000), mouse anti-Flag (M2, Sigma, 1:2000), mouse anti-panCadherin (CH-19, Sigma, 1:4000), rabbit anti-Flag (Sigma, 1:4000), rabbit anti-GFP (Invitrogen, 1:2000) and horseradish peroxydase-conjugated secondary antibodies (Jackson Immunoresearch).

### Immunostaining

Twenty-four hours after transfection, cells were fixed in 4% paraformaldehyde for 20 min. Immunostaining was performed using mouse anti-HA (16B12, Convance, 1:2000), rabbit anti-cleaved-Caspase-3 (Cell Signaling Technology, 1:1000) and secondary antibodies coupled to alexa488 (Invitrogen), Cy3 or Cy5 (Jackson Immunoresearch). 4′,6-diamidino-2-phenylindole (DAPI) was used for nuclear staining. Surface HA immunostaining was performed in the complete absence of permeabilizing agent using a secondary antibody coupled to Cy3, followed by total HA immunostaining in the presence of detergents and revealed with a secondary antibody conjugated to Cy5. Images were acquired using a Zeiss LSM710 confocal microscope.

### TCGA data mining and analysis

Legacy data from TCGA were obtained from the NCI Genomic Data Commons^34^. Analyses were performed using the software R (v 3.5.1) and the *TCGAbiolinks* package (v 2.8.4)^35^.

### Statistics

The percentage of Caspase-3^+^ cells among HA^+^ cells was counted from at least 500 cells obtained from a minimum of three independent experiments. Means were compared using Student’s *t*-test and non-parametric Mann–Whitney test. Comparison of gene expression levels in paired tumor and control samples was achieved using the non-parametric Wilcoxon test. Survival curves were compared using the non-parametric log-rank test. Statistical results are reported in Supplementary Tables 1, 2.

## Supplementary information

Supplementary figure 1

Supplementary table 1

Supplementary table 2

Supplementary table 3

Supplementary table 4

Supplementary table 5

Supplemental Material File #1

## References

[1] Goldschneider D, Mehlen P (2010). Dependence receptors: a new paradigm in cell signaling and cancer therapy. Oncogene.

[2] Mehlen P (1998). The DCC gene product induces apoptosis by a mechanism requiring receptor proteolysis. Nature.

[3] Llambi F, Causeret F, Bloch-Gallego E, Mehlen P (2001). Netrin-1 acts as a survival factor via its receptors UNC5H and DCC. EMBO J..

[4] Delloye-Bourgeois C (2013). Sonic Hedgehog promotes tumor cell survival by inhibiting CDON pro-apoptotic activity. PLoS Biol..

[5] Luchino J (2013). Semaphorin 3E suppresses tumor cell death triggered by the plexin D1 dependence receptor in metastatic breast cancers. Cancer Cell..

[6] Rabizadeh S (1993). Induction of apoptosis by the low-affinity NGF receptor. Science.

[7] Lin S (2017). Non-canonical NOTCH3 signalling limits tumour angiogenesis. Nat. Commun..

[8] Wang H (2018). The Proto-oncogene c-Kit inhibits tumor growth by behaving as a dependence receptor. Mol. Cell..

[9] Gibert B, Mehlen P (2015). Dependence receptors and cancer: addiction to trophic ligands. Cancer Res..

[10] Fitamant J (2008). Netrin-1 expression confers a selective advantage for tumor cell survival in metastatic breast cancer. Proc. Natl Acad. Sci. USA.

[11] Delloye-Bourgeois C (2009). Interference with netrin-1 and tumor cell death in non-small cell lung cancer. J. Natl. Cancer Inst..

[12] Delloye-Bourgeois C (2009). Netrin-1 acts as a survival factor for aggressive neuroblastoma. J. Exp. Med..

[13] Grandin M (2016). Structural decoding of the Netrin-1/UNC5 interaction and its therapeutical implications in cancers. Cancer Cell.

[14] Causeret F, Sumia I, Pierani A (2016). Kremen1 and Dickkopf1 control cell survival in a Wnt-independent manner. Cell Death Differ..

[15] Glinka A (1998). Dickkopf-1 is a member of a new family of secreted proteins and functions in head induction. Nature.

[16] Galluzzi L (2012). Molecular definitions of cell death subroutines: recommendations of the nomenclature committee on cell death 2012. Cell Death Differ..

[17] Nakamura T, Nakamura T, Matsumoto K (2008). The functions and possible significance of Kremen as the gatekeeper of Wnt signalling in development and pathology. J. Cell. Mol. Med..

[18] Wang JJ (2000). Dimerization-dependent block of the proapoptotic effect ofp75(NTR). J. Neurosci. Res..

[19] Mille F (2009). Interfering with multimerization of netrin-1 receptors triggers tumor cell death. Cell Death Differ..

[20] Mao B (2002). Kremen proteins are Dickkopf receptors that regulate Wnt/beta-catenin signalling. Nature.

[21] Hassler C (2007). Kremen is required for neural crest induction in Xenopus and promotes LRP6-mediated Wnt signaling. Development.

[22] Lu H, Ma J, Yang Y, Shi W, Luo L (2013). EpCAM is an endoderm-specific Wnt derepressor that licenses hepatic development. Dev. Cell..

[23] Davidson G, Mao B, del Barco Barrantes I, Niehrs C (2002). Kremen proteins interact with Dickkopf1 to regulate anteroposterior CNS patterning. Development.

[24] Kimura-Yoshida C, Mochida K, Ellwanger K, Niehrs C, Matsuo I (2015). Fate specification of neural plate border by canonical Wnt signaling and Grhl3 is crucial for neural tube closure. EBioMedicine.

[25] Nakamura T (2001). Molecular cloning and characterization of Kremen, a novel kringle-containing transmembrane protein. Biochim. Biophys. Acta.

[26] Osada M (2006). The Wnt signaling antagonist Kremen1 is required for development of thymic architecture. Clin. Dev. Immunol..

[27] Wu D, Murashov AK (2013). MicroRNA-431 regulates axon regeneration in mature sensory neurons by targeting the Wnt antagonist Kremen1. Front. Mol. Neurosci..

[28] Pan L (2019). Higher-order clustering of the transmembrane anchor of DR5 drives signaling. Cell.

[29] Lemmon MA, Schlessinger J (2010). Cell signaling by receptor tyrosine kinases. Cell.

[30] Siebold C, Jones EY (2013). Structural insights into semaphorins and their receptors. Semin. Cell Dev. Biol..

[31] Sakamoto K, Chao WS, Katsube K, Yamaguchi A (2005). Distinct roles of EGF repeats for the Notch signaling system. Exp. Cell Res..

[32] Vilar M (2009). Activation of the p75 neurotrophin receptor through conformational rearrangement of disulphide-linked receptor dimers. Neuron.

[33] Lin Z (2015). Structural basis of death domain signaling in the p75 neurotrophin receptor. Elife.

[34] Grossman RL (2016). Toward a shared vision for cancer genomic data. N. Engl. J. Med..

[35] Colaprico A (2016). TCGAbiolinks: an R/Bioconductor package for integrative analysis of TCGA data. Nucleic Acids Res..

