# Supplementary figures and images for: Kremen1-induced cell death is regulated by homo- and heterodimerization
Source: Cell Death Discov. 2019 May 1;5:91. doi: 10.1038/s41420-019-0175-5 (PMC6494814; doi:10.1038/s41420-019-0175-5)

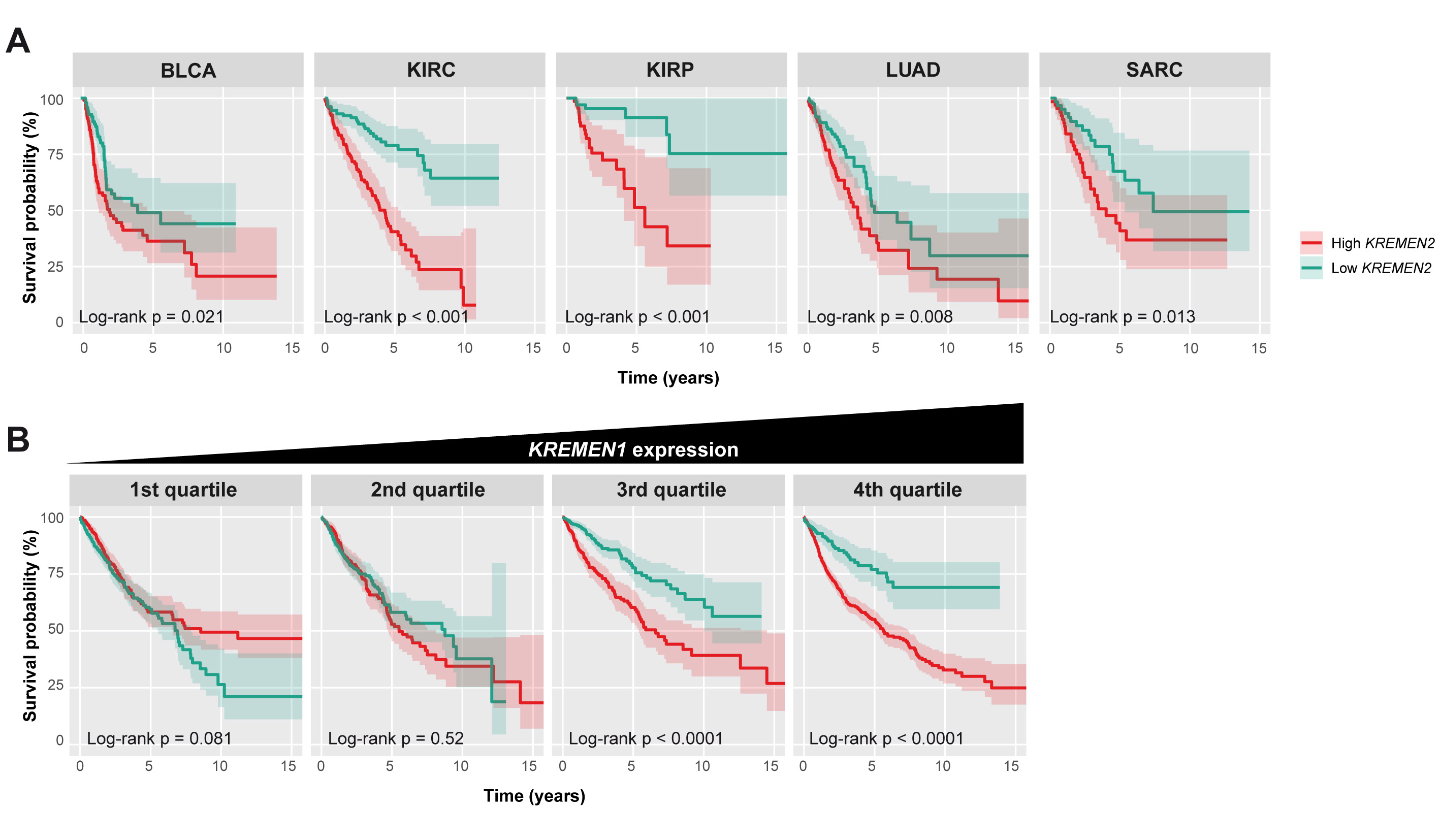

Supplement: Supplementary file 1 — Supplementary figure 1 [file 41420_2019_175_MOESM1_ESM.jpg]
