# Supplementary material for: Kremen1-induced cell death is regulated by homo- and heterodimerization
Source: Cell Death Discov. 2019 May 1;5:91. doi: 10.1038/s41420-019-0175-5 (PMC6494814; doi:10.1038/s41420-019-0175-5)
Supplement: Supplementary file 2 — Supplementary table 1 [file 41420_2019_175_MOESM2_ESM.pdf]

	Fig 2B		Fig 2C		Fig 4B				
condition	GFP	Krm1secECD	Ctrl	AP20187	GFP	Krm2	Krm2ΔECD	Krm2ΔICD	Krm2secECD
number of cells	702	707	1014	909	1427	1660	1393	1076	732
number of replicates	4	4	8	8	11	10	9	6	4
t-test p value		0.00006		0.00058		0.00000	0.03226	0.00002	0.00023
Mann Whitney p value		0.02860		0.00470		<0.0001	0.13080	0.00020	0.00150

p < 0,001
0,01 < p < 0,05
p < 0,05
