# Supplementary material for: Kremen1-induced cell death is regulated by homo- and heterodimerization
Source: Cell Death Discov. 2019 May 1;5:91. doi: 10.1038/s41420-019-0175-5 (PMC6494814; doi:10.1038/s41420-019-0175-5)
Supplement: Supplementary file 3 — Supplementary table 2 [file 41420_2019_175_MOESM3_ESM.pdf]

Cancer type	TCGA-BLCA	TCGA-BRCA	TCGA-CHOL	TCGA-COAD	TCGA-ESCA	TCGA-HNSC	TCGA-KICH	TCGA-KIRC	TCGA-KIRP
Kremen1 p-value	0.332065582	8.66E-16	0.01953125	3.76E-05	0.174804688	0.000434366	0.001815677	1.62E-07	1.12E-05
Dkk1 p-value	0.000335693	0.911038338	0.02734375	0.009382546	0.1015625	3.85E-06	0.006129205	0.730004798	0.002564962
Kremen2 p-value	0.007144928	7.34E-19	0.00390625	9.15E-06	0.1015625	1.17E-06	0.00080812	3.87E-08	8.26E-06
n	19	113	9	26	11	43	25	72	32
Kremen1 fold change	0.96	0.54	2.32	0.55	0.43	0.67	1.77	0.65	0.43
Dkk1 fold change	0.31	0.84	3.55	1.89	3.96	5.62	0.19	0.99	0.46
Kremen2 fold change	5.01	4.50	11.26	2.60	7.54	3.85	2.00	2.01	2.50

p < 0,05

0,01 < p < 0,05

p < 0,001

Significantly downregulated

Significantly upregulated

TCGA-LIHC	TCGA-LUAD	TCGA-LUSC	TCGA-PAAD	TCGA-PRAD	TCGA-READ	TCGA-STAD	TCGA-THCA	TCGA-UCEC
0.016231578	0.81632797	1.82E-09	0.875	0.047618825	0.15625	0.963233216	6.49E-06	0.8125
0.007454617	0.692946398	0.000832847	0.375	0.001704925	0.15625	0.119450366	0.002904423	0.15625
0.000235716	1.60E-10	7.14E-10	0.625	0.176254701	0.21875	8.19E-05	6.32E-09	1
50	58	51	4	52	6	32	59	7
1.43	0.96	3.02	1.07	0.88	1.82	0.94	0.77	0.30
2.01	0.71	2.66	64.44	0.44	24.82	2.25	1.43	2.59
1.90	3.38	34.89	1.38	0.63	2.15	3.37	2.25	1.36
