# Supplementary material for: Kremen1-induced cell death is regulated by homo- and heterodimerization
Source: Cell Death Discov. 2019 May 1;5:91. doi: 10.1038/s41420-019-0175-5 (PMC6494814; doi:10.1038/s41420-019-0175-5)
Supplement: Supplementary file 4 — Supplementary table 3 [file 41420_2019_175_MOESM4_ESM.pdf]

BLCA	Bladder Urothelial Carcinoma
BRCA	Breast invasive carcinoma
CHOL	Cholangiocarcinoma
COAD	Colon adenocarcinoma
ESCA	Esophageal carcinoma
HNSC	Head and Neck squamous cell carcinoma
KICH	Kidney Chromophobe
KIRC	Kidney renal clear cell carcinoma
KIRP	Kidney renal papillary cell carcinoma
LIHC	Liver hepatocellular carcinoma
LUAD	Lung adenocarcinoma
LUSC	Lung squamous cell carcinoma
PAAD	Pancreatic adenocarcinoma
PRAD	Prostate adenocarcinoma
READ	Rectum adenocarcinoma
SARC	Sarcoma
STAD	Stomach adenocarcinoma
THCA	Thyroid carcinoma
UCEC	Uterine Corpus Endometrial Carcinoma
