# Supplementary material for: Kremen1-induced cell death is regulated by homo- and heterodimerization
Source: Cell Death Discov. 2019 May 1;5:91. doi: 10.1038/s41420-019-0175-5 (PMC6494814; doi:10.1038/s41420-019-0175-5)
Supplement: Supplementary file 5 — Supplementary table 4 [file 41420_2019_175_MOESM5_ESM.pdf]

	logFC	FDR	Normal	Tumor	Delta
KRT17	6.82	2.75E-39	263.2	27562.8	1795.4
TOP2A	4.67	3.03E-73	119.1	2603.9	555.9
TP63	5.76	1.38E-40	82.4	4128.5	474.8
EEF1A2	4.81	2.13E-39	83.6	1964.1	402.6
MMP11	5.03	7.94E-74	75.5	2252.2	379.5
FOXM1	4.68	6.18E-94	62.6	1361.9	293.1
PI3	6.58	1.86E-39	40.7	3332.6	267.9
NPTX2	5.04	3.69E-41	49.6	1040.6	249.9
LYPD3	5.31	3.78E-54	43.5	1635.8	231.1
MYBL2	4.84	4.22E-70	40.9	1021.3	198.0
CA9	5.81	4.62E-39	32.5	1080.9	188.6
CDC20	4.60	1.30E-80	37.9	805.9	174.3
PTHLH	6.20	3.16E-57	21.8	1324.4	135.2
UBE2C	4.85	8.41E-95	27.8	666.7	134.6
IGF2BP3	4.85	1.99E-82	21.4	514.7	103.8
SRRM3	4.93	2.74E-62	20.4	548.5	100.4
KIF4A	4.72	1.98E-95	20.1	456.1	94.7
BIRC5	4.76	3.91E-83	18.7	439.2	89.1
COL11A1	6.28	1.08E-38	14.2	1024.2	89.0
ATP1A3	6.54	1.45E-45	13.4	1055.9	87.8
KIF1A	5.63	5.90E-48	14.1	548.7	79.4
HJURP	4.69	6.28E-84	15.8	339.8	74.2
MELK	4.81	3.17E-80	15.0	365.5	72.4
HCN2	5.57	2.20E-48	12.6	568.1	70.3
DLGAP5	4.75	1.57E-69	14.7	349.8	70.1
FAM83A	6.32	5.28E-40	10.8	735.9	68.5
NEK2	4.74	1.34E-88	14.4	321.5	68.1
TROAP	4.83	1.26E-95	13.5	322.0	65.1
CDC45	4.77	1.42E-76	13.5	302.5	64.5
KIF18B	4.92	2.50E-84	12.7	320.3	62.6
TGM3	5.34	1.89E-41	11.6	462.7	61.9
NXPH4	5.63	2.10E-76	10.6	501.5	59.8
NUF2	4.66	7.85E-74	12.0	270.7	55.9
DEPDC1	4.60	1.03E-80	12.1	247.7	55.4
TTK	4.87	4.71E-80	11.3	294.6	55.2
PITX1	6.31	7.62E-53	8.6	636.6	54.5
KIF14	4.61	5.98E-93	11.6	224.9	53.7
CABYR	4.68	4.58E-65	11.0	254.8	51.7
GRHL3	4.58	3.99E-38	11.0	237.7	50.3
EXO1	4.60	9.66E-83	9.8	203.2	45.1
PPP2R2C	6.21	7.04E-54	7.1	499.9	44.2
GJB5	5.28	6.83E-42	8.3	288.7	43.7
PRAME	6.82	2.62E-44	6.2	662.1	42.0
AFAP1-AS1	5.59	2.02E-43	7.4	327.7	41.4
E2F7	4.65	1.11E-64	8.4	185.0	38.9
B4GALNT4	6.14	1.63E-52	5.8	342.6	35.7
CENPA	4.82	6.19E-87	6.2	150.9	30.0
GJB6	8.39	6.86E-44	3.5	1068.5	29.1
CST1	7.75	1.41E-39	3.4	643.0	26.4
HMGA2	7.25	2.75E-52	3.2	458.2	22.9

RNASE7	5.50	1.08E-53	4.0	130.4	22.2
CDC25C	4.94	2.68E-97	4.0	106.8	19.9
DLX5	6.32	1.02E-43	3.1	230.1	19.4
ZIC2	5.37	1.50E-43	3.3	132.0	17.5
OTX1	5.01	1.46E-39	3.5	102.3	17.4
BARX1	5.99	3.95E-38	2.3	87.0	13.7
MLANA	5.34	1.75E-38	2.5	101.3	13.3
NEIL3	4.58	1.26E-72	2.9	62.3	13.2
KLK4	8.82	2.09E-40	1.4	645.2	12.7
FAM83C	7.22	6.77E-44	1.5	178.3	11.2
**KREMEN2**	**7.04**	**3.42E-64**	**1.6**	**186.0**	**11.1**
KCNN1	5.33	8.11E-46	1.9	67.8	10.4
SHOX2	5.35	2.77E-53	1.8	63.8	9.5
CERS3	7.95	9.12E-43	1.1	241.5	8.4
PNMA5	7.30	5.41E-39	1.1	158.4	8.2
HHIPL2	6.70	2.82E-41	1.2	148.8	8.2
SLC39A2	4.66	6.83E-42	1.7	34.4	8.1
MAGEA6	9.09	3.82E-38	0.8	406.1	7.5
FOXH1	4.84	3.27E-49	1.3	40.3	6.5
ZNF695	4.64	4.61E-49	1.4	32.6	6.4
C1orf61	5.25	1.49E-45	1.2	36.8	6.1
DQX1	5.76	3.41E-42	1.1	54.5	6.1
SMC1B	4.94	1.36E-51	1.0	26.4	4.9
LINC00669	4.87	3.46E-56	0.9	23.3	4.2
DLX6	7.56	1.26E-39	0.5	99.5	3.7
HOXA13	6.26	6.35E-67	0.6	45.5	3.5
C5orf46	5.48	7.88E-57	0.6	23.7	3.2
HOXC13	6.84	5.83E-42	0.4	54.6	2.8
EN1	6.97	6.78E-42	0.4	49.8	2.7
DLX1	6.02	1.44E-51	0.4	26.4	2.5
GABRA3	6.87	1.20E-40	0.3	46.6	2.3
TERT	6.88	3.76E-49	0.2	37.9	1.6
DLX2	5.51	1.77E-40	0.2	13.1	1.3
DMBX1	6.46	3.34E-38	0.1	18.2	0.9
MAGEA3.1	9.09	3.82E-38	NA	NA	NA
