# Supplementary material for: Kremen1-induced cell death is regulated by homo- and heterodimerization
Source: Cell Death Discov. 2019 May 1;5:91. doi: 10.1038/s41420-019-0175-5 (PMC6494814; doi:10.1038/s41420-019-0175-5)
Supplement: Supplementary file 6 — Supplementary table 5 [file 41420_2019_175_MOESM6_ESM.pdf]

	logFC	FDR	Normal	Tumor	Delta
SLC2A1	3.00	2.85E-58	1138.7	8475.3	3421.1
TOP2A	3.35	3.47E-75	298.7	2701.8	1000.5
MMP11	3.30	1.89E-49	301.7	3005.8	996.6
TPX2	3.30	1.91E-91	188.1	1565.6	621.1
CENPF	3.22	1.62E-82	175.9	1476.6	567.4
MMP1	4.80	3.55E-58	100.8	2545.9	483.7
FOXM1	3.31	3.21E-85	140.5	1244.3	465.2
MYBL2	3.07	7.54E-51	145.9	1095.9	447.7
ANLN	2.92	9.23E-58	146.1	960.8	426.7
ETV4	3.28	7.71E-85	88.5	765.7	290.4
UBE2C	3.27	3.87E-67	86.2	724.6	282.0
PMEL	3.44	1.80E-51	81.5	843.7	280.7
NPTX2	4.17	6.94E-59	62.4	908.0	260.4
COL10A1	3.69	1.13E-45	67.8	868.3	249.9
IQGAP3	3.07	5.54E-69	74.3	551.4	227.8
ASPM	3.35	6.90E-81	66.2	597.0	221.7
CEP55	2.92	2.68E-58	70.7	471.8	206.4
KIF2C	3.00	1.06E-70	68.7	495.6	206.2
CDC6	3.06	2.99E-89	67.4	497.3	206.2
KIF20A	2.98	7.19E-67	64.9	461.3	193.7
COL11A1	5.14	2.59E-55	37.5	1328.0	192.6
TRIP13	2.99	1.69E-78	61.5	446.9	184.0
FBN2	3.44	1.94E-49	52.9	558.9	182.0
BIRC5	3.01	1.41E-58	55.9	391.6	168.4
BUB1B	2.95	1.97E-69	54.6	380.5	161.2
KIF4A	3.35	9.70E-84	47.1	433.4	157.8
NCAPH	2.92	5.65E-69	51.6	350.8	151.0
DLGAP5	3.01	1.64E-53	47.7	341.9	143.5
PKMYT1	2.92	3.81E-65	47.9	314.7	140.0
MELK	3.22	1.39E-66	43.2	355.2	139.0
NXPH4	3.17	5.03E-47	40.6	354.5	128.6
CDKN2A	3.86	3.60E-101	32.3	410.7	124.6
HJURP	3.32	8.47E-79	37.3	331.5	123.8
NEK2	3.33	1.84E-74	36.7	328.2	122.2
TTK	3.10	2.86E-66	37.8	287.9	117.1
IGF2BP3	4.07	2.07E-96	28.7	423.2	116.7
GTSE1	2.95	4.27E-73	36.9	253.7	108.5
TROAP	3.29	4.42E-76	32.9	283.5	108.2
KIF18B	3.43	5.85E-74	31.3	296.8	107.4
CDC45	3.19	2.91E-64	32.5	258.9	103.8
NUF2	3.31	5.73E-74	29.7	262.8	98.4
PRAME	5.23	2.19E-48	18.0	681.5	94.0
GRIN2D	2.98	8.23E-59	29.9	208.9	88.9
TICRR	2.99	7.36E-67	29.0	202.7	86.7
CKAP2L	3.04	3.43E-70	28.0	201.9	84.9
KIF14	3.50	1.81E-88	22.5	225.0	78.7
CDCA2	2.99	3.14E-64	26.1	183.5	78.1
CABYR	3.18	1.40E-60	24.5	217.0	77.7
MCM10	3.24	4.78E-74	23.9	198.4	77.5
EXO1	3.31	1.83E-82	22.7	200.9	75.0

RAD54L	3.04	5.33E-78	24.3	179.4	74.0
POLQ	3.17	5.65E-72	22.5	179.5	71.4
MMP13	6.75	6.84E-52	10.3	1140.1	69.4
HCN2	4.37	4.29E-65	14.9	276.4	65.0
E2F7	3.44	9.87E-77	17.5	170.7	60.2
CENPA	3.21	2.81E-69	18.0	147.6	57.6
HMGA2	4.89	8.65E-64	11.1	317.4	54.4
CDC25C	2.97	1.14E-58	12.9	92.0	38.4
**KREMEN2**	**4.17**	**6.16E-58**	**8.2**	**132.6**	**34.3**
OTX1	3.64	7.88E-50	8.4	90.7	30.6
CENPI	3.02	6.90E-68	9.0	66.7	27.2
ZIC2	4.20	4.32E-48	6.5	107.6	27.2
NEIL3	2.93	3.31E-54	8.3	57.0	24.4
SLC30A3	3.62	4.60E-51	4.5	56.3	16.2
KCNN1	3.51	1.81E-50	3.7	38.4	13.1
ZNF695	3.35	5.55E-66	3.8	36.9	12.9
GRM4	5.19	8.52E-62	2.4	80.4	12.4
PNMA5	6.71	1.00E-64	1.4	153.7	9.4
SMC1B	4.53	6.24E-77	1.6	35.8	7.4
DLL3	4.40	2.49E-46	1.5	30.4	6.6
TERT	5.23	3.89E-49	1.2	48.0	6.4
DPF1	3.29	1.99E-48	1.8	16.6	6.0
TCAM1P	4.06	8.42E-46	1.1	17.6	4.3
PLAC1	3.76	1.71E-48	1.1	14.3	4.3
DMBX1	5.20	4.03E-54	0.7	26.7	3.4
FEZF1	4.95	2.47E-47	0.6	19.6	3.1
KISS1R	4.21	1.65E-61	0.7	12.7	2.8
TEX19	5.16	6.25E-57	0.4	16.0	2.2
GNGT1	3.89	9.22E-46	0.3	6.0	1.4
GBX2	3.85	1.20E-45	0.3	5.0	1.1
