# Supplementary material for: Kremen1-induced cell death is regulated by homo- and heterodimerization
Source: Cell Death Discov. 2019 May 1;5:91. doi: 10.1038/s41420-019-0175-5 (PMC6494814; doi:10.1038/s41420-019-0175-5)
Supplement: Supplementary file 7 — Supplemental Material File #1 [file 41420_2019_175_MOESM7_ESM.doc]

**Supplementary Figure 1. Increased survival of patients with low *KREMEN2* expression in tumors.**

(A) Survival curves for cancer types showing a significantly better outcome for patients with low *KREMEN2* expression in tumors. (B) Survival curves for patient of all cancer except the five shown in (A), ranked by the level of *KREMEN1* expression in tumors. The link between *KREMEN1*, *KREMEN2* and survival is not due to a bias in sampling.

**Supplementary Table 1. Caspase3^+^ cells quantifications.**

Table indicating for each condition: the total number of cells considered, the number of replicate experiments and the p value using either Student’s t test or the non parametric Mann-Whitney test (compared to control). The significance is color coded according to the legend.

**Supplementary Table 2. Statistics regarding expression in cancers.**

Related to Figure 5A-C. Table indicating for each cancer type (refer to Supplementary table 3 for abbreviations) the statistical significance (Wilcoxon test) between paired control and tumor samples for the expression of *Kremen1*, *Dkk1* and *Kremen2*. The number of patients is indicated (only patients for which a control sample was available were taken into account). The fold change corresponds to the average of each patient’s ratio between tumor expression and normal tissue expression. Both the significance and fold change are color coded, according to the legend.

**Supplementary Table 3. Abbreviations of TCGA cancer studies.**

Related to Figure 5. List of the TCGA cancer studies abbreviations used in this study.

**Supplementary Table 4. Most significantly upregulated genes in CHOL, KICH, LIHC and LUSC.**

Related to Figure 5D. List of 85 genes corresponding to the intersection between the top 500 genes ranked by fold change (most upregulated) and the bottom 500 ranked by FDR (most significantly differentially expressed) when comparing tumor and control samples from CHOL, KICH, LIHC and LUSC (cancer types showing increased *KREMEN1* expression in tumors relative to control tissue).

**Supplementary Table 5. Most significantly upregulated genes in tumor samples showing increased *KREMEN1* expression.**

Related to Figure 5E. List of 80 genes corresponding to the intersection between the top 500 genes ranked by fold change (most upregulated) and the bottom 500 ranked by FDR (most significantly differentially expressed) when considering tumor and control samples showing increased *KREMEN1* expression in tumors, regardless of the cancer type.
